# Developmental features of sleep electrophysiology in family dogs

**DOI:** 10.1038/s41598-021-02117-1

**Published:** 2021-11-23

**Authors:** Vivien Reicher, Nóra Bunford, Anna Kis, Cecília Carreiro, Barbara Csibra, Lorraine Kratz, Márta Gácsi

**Affiliations:** 1 Department of Ethology, Institute of Biology, Eötvös Loránd University, Pázmány Péter sétány 1/C, Budapest, 1117 Hungary; 2 MTA-ELTE Comparative Ethology Research Group, Budapest, Hungary; 3 Developmental and Translational Neuroscience Research Group, Institute of Cognitive Neuroscience and Psychology, Research Centre for Natural Sciences, Budapest, Hungary; 4 Institute of Cognitive Neuroscience and Psychology, Research Centre for Natural Sciences, Budapest, Hungary

**Keywords:** Developmental biology, Neuroscience, Development of the nervous system

## Abstract

Age-related differences in dog sleep and the age at which dogs reach adulthood as indexed by sleep electrophysiology are unknown. We assessed, in (1) a Juvenile sample (*n* = 60) of 2–14-month-old dogs (weight range: 4–68 kg), associations between age, sleep macrostructure, and non-rapid eye movement (NREM) EEG power spectrum, whether weight moderates associations, and (2) an extended sample (*n* = 91) of 2–30-months-old dogs, when sleep parameters stabilise. In Juvenile dogs, age was positively associated with time in drowsiness between 2 and 8 months, and negatively with time in rapid eye movement (REM) sleep between 2 and 6 months. Age was negatively associated with delta and positively with theta and alpha power activity, between 8 and 14 months. Older dogs exhibited greater sigma and beta power activity. Larger, > 8-month-old dogs had less delta and more alpha and beta activity. In extended sample, descriptive data suggest age-related power spectrum differences do not stabilise by 14 months. Drowsiness, REM, and delta power findings are consistent with prior results. Sleep electrophysiology is a promising index of dog neurodevelopment; some parameters stabilise in adolescence and some later than one year. Determination of the effect of weight and timing of power spectrum stabilisation needs further inquiry. The dog central nervous system is not fully mature by 12 months of age.

## Introduction

Sleep is a relatively immobile, reversible state, characterised by absent or limited responsiveness to the external environment (without loss of consciousness) and is regulated by circadian and homeostatic processes^1^. It is a complex combination of behavioural and physiological processes that affects several aspects of cognitive development^2^ as well as dimensions of functioning^3^. Findings across studies indicate that sleep plays a role in animal (neuro)development (for review, see^2,4^).

Sleep is characterised by the ultradian rhythms of two distinct forms of electroencephalographic (EEG) activity: desynchronised, rapid waveforms with a small amplitude constitute rapid eye movement (REM) sleep, and synchronised, slow waveforms with high amplitude constitute non-REM (NREM) sleep. In humans, the first sleep stage following wakefulness is NREM 1, during which alpha waves (8–12 Hz) characteristic of quiet wakefulness cease and low-voltage theta activity (4–8 Hz) appears. The second stage, NREM 2, is characterised by theta and delta (1–4 Hz) activity and/or sleep spindles (sigma activity bursts, 12–16 Hz). In the third stage, also called slow wave sleep (SWS), high-voltage delta frequency waveforms are dominant. NREM is followed by REM, characterised by mixed theta and beta activity, rapid saccadic eye movements and muscle atonia^5^.

This description of basic sleep architecture is applicable to humans and to other mammals, though there are some inter-species differences. The transition from wakefulness to sleep, for example, is not as clear in carnivore and insectivore mammals^6,7^, as in humans^5^. These species exhibit drowsiness, a sleep stage that bears characteristics of both human NREM 1 sleep and quiet wakefulness. Different levels of NREM sleep are also not as differentiated in other species as in humans, but constitute one universal stage, slow wave sleep^8^ (though more recent findings indicate this might be a methodological issue^9^). Yet another difference is related to NREM/REM cycles. In most cases in humans, REM is followed by NREM sleep, whereas other mammals are more likely to wake up after REM sleep, potentially reflecting an evolutionary adaptation to limit time spent in a vulnerable state^10^.

Developmental changes as reflected by neural indices of sleep are not specific to humans (for review see^2^) but are commonly observed across many mammalian species^11^. For example, in several species, there is longer total sleep duration during early, relative to later developmental stages (human^12^; rat, cat, guinea pig^13^). Furthermore, in the first month of life, a decrease in REM sleep and an increase in NREM sleep has been observed in the cat, rat and guinea pig^13,14^. It has been proposed that REM sleep may serve as an endogenous source of activation that supports neurodevelopment, e.g., in the form of muscle twitches during sleep^15^ and this may explain why there is an age-related decrease in this sleep parameter. As aging proceeds, changes in sleep architecture are less pronounced during pubertal development: for example, in rats, there is a decrease in both REM and NREM sleep accompanied by an increase in wakefulness^14^. To our knowledge, developmental changes in sleep parameters have not been documented in non-human primates, except for cross-sectional comparisons of young adult and aged animals, e.g., in rhesus monkeys^16^ or in mouse lemurs^17^.

Developmental changes are not only reflected in age-related alterations in sleep macrostructure but also in EEG spectrum. Given their assumed role in cortical maturation, the majority of available, pertinent developmental studies are focused on delta power (1–4 Hz) and/or slow wave activity (SWA; 1–4.5 Hz) changes during NREM sleep (e.g.^14,18^). Both rat and human data show that the activity of slow waves increases during the first years of life, reaches a maximum by puberty, and declines throughout adolescence (rat^14^; human^19,20^). These changes likely reflect synaptic pruning^18,21^. A similar pattern has been found in dogs, using invasive methods^22^.

Taken together, neurodevelopmental changes mirrored by differences in distinct aspects of sleep are observable across some mammalian species. Yet, not just with regard to development but more generally, it is a notable limitation to the literature that available findings on animal sleep have been mostly obtained with methods that limit their comparability across humans and nonhuman animals. Specifically, in available animal studies, invasive manipulations and techniques, such as drugs, lesions and/or sensory deprivations have been used (see review^4^). Given differences between chemically-induced and natural sleep^23^ and that intracranial electrodes can only be used with a restricted subgroup of animals (i.e., laboratory-bred and -kept animals), the generalizability of these results is limited, underscoring need for animal models with which sleep can be studied naturalistically, using non-invasive methods.

Relatedly, the family dog has been recognised as unique among domesticated species, because both its evolutionary history and shared social environment with humans have contributed to changes in dog socio-cognitive skills^24,25^. Regarding sleep research specifically, dogs have been repeatedly shown to be an ideal model species for non-invasive sleep EEG studies^7,26–30^. The general architecture of human sleep is better approximated by dog sleep than by that of commonly used laboratory animals. For example, the primary diurnal sleep phase in humans and dogs is in the dark, whereas that of mice and rats is in the light; the daily sleep duration in humans is around 8 h, in dogs it is 8–14 h, while in mice, rats, and cats it is longer, around 12–15 h^31^. Although early studies on dog sleep were focused on neurological conditions (e.g., epilepsy) and involved invasive methods with laboratory animals^32,33^, more recent investigations capitalised upon the advantages of this species, by using a non-invasive polysomnography method^7^. This novel method has been successfully implemented in several studies assessing the basic architecture of dog sleep (i.e., in the absence of pre-sleep active experiences and/or handling)^7,34,35^ and the correspondence between awake functioning, sleep architecture and pre-sleep activities including learning^27^ and emotional exposure^28^ as well as timing and location of sleep^26^. Accordingly, the dog is a promising model for comparative sleep research and findings indicating age-related changes and differences in dog behaviour indicate it may also be suitable to model developmental differences reflected in neural indices of sleep.

Available data suggest developmental differences in dogs’ socio-cognitive abilities, this concerns for example age-related differences in how well dogs follow human pointing gestures, with lower performance in 10–12-month olds relative to younger (2–10) and older (12–14) dogs^36^. Reports on similar abilities in puppies and adult dogs can also be found, for example, social referencing seems to develop early (puppies^37,38^; adults^39,40^). Executive functions, such as working memory, were observed in puppies of 8–10 weeks old^41^, but less developed than in dogs above 1 year old^42^.

Morphological and structural brain changes in dogs have also been described but only with regard to very young animals (8–36 weeks)^43^ or in old dogs (> 7 years)^44^. Beyond these studies, the majority of the literature examining age-related changes in dog behaviour (e.g.^45^), cognition (e.g.^46^), brain anatomy (e.g.,^47^) or neural processes (e.g.,^7^), consists of studies with ‘adult’, i.e., 12-month-old or older animals or declare that dogs older than 12 months of age are adults (e.g.^48^). Yet others focused on changes throughout the lifespan but not explicitly on early development (despite having the data to do so), with such focus potentially obscuring age-related differences in very young animals (e.g.^49^). Despite notable absence of empirical data on when brain maturation in dogs may mark the onset of/transition into adulthood as well as evidence that dogs from any size category reach their final weight by 12 months^50^, it is a generally accepted (unspoken) assumption in the literature that dogs over one year of age are adults.

In addition to age, differences in morphological features across dogs and differences in developmental rates between breeds may influence EEG data. Regarding the former, dogs present significant intraspecific variability with regard to head musculature and skull shape and thickness. To circumvent measurement error that might arise as a result of these differences, dog EEG data are analysed using not absolute, but relative EEG power^7^, as in human studies^51^. Certain breeds may mature at a faster rate than others. For example, dogs with greater muscle and/or skull thickness are suggested to develop at a slower rate and reach sexual maturity later, given hormonal^52^ and sexual^53,54^ changes. As the muscle and skull thickness of a dog is typically associated with their adult weight, differences in developmental status might be indexed as a dog’s adult weight. For example, when comparing beagles and great Danes, basal plasma growth hormone (GH) increases in both breeds until the age of 7 weeks, but high GH release persisted only in great Danes until the age of 24 weeks^52^. Further, in fox terriers, a breed with an average adult weight of 8 kg, the first spermatozoa in the ejaculate occurs when the animals are between 8 and 9 months old^53^ whereas in Collies, a breed with an average adult weight of 23 kg, the first spermatozoa are observed when the animals are between 11 and 12 months old^54^.

Taken together, evidence in both humans and non-human animals indicates that developmental differences and, as such, developmental status, may be reflected in parameters of sleep. However, the majority of available findings have been obtained using invasive methods and performed mainly on what are assumed to be adult animals. Thus, developmental differences in specific aspects of animal sleep or when such differences stabilise (and thus indicate maturity) are unknown.

For the present study, we assumed that age-related differences in the sleep architecture of dogs are generally similar to corresponding differences in other mammals. To test this, we analysed age-related differences in sleep macrostructure (drowsiness, NREM and REM) and spectral power (delta 1–4 Hz, theta 4–8 Hz, alpha 8–12 Hz, sigma 12–16 Hz, beta 16–30 Hz) variables. Weight was also included in our analyses.

## Methods

### Subjects

Altogether, data from 91, 2–30-month-old family dogs were analysed. First, to determine whether sleep parameters stabilise by the time dogs reach the age of 1 year, data were collected from 60, 2–14-month-old dogs (Juvenile sample) (*Mean* age = 7.8, *SD* = 4.0). This consisted of 50 purebreds from 25 different breeds and 10 mongrels; 32 females (*Mean* age = 8, *SD* = 4.0; of the 32 females, 15 were neutered and for 1, there was no such information available) and 28 males (*Mean* age = 7.7, *SD* = 4.2; out of the 28 male dogs 3 were neutered). Second, to assess whether sleep parameters change beyond the age of 14 months, we extended our sample (and thus, the age range thereof) by adding data from our previous dog sleep EEG studies^7,26–29^, so that the total sample consisted of 91 family dogs (Extended sample encompassing the Juvenile sample + *n* = 31, > 14 month-old dogs) with an age range of 2–30 months. This sample consisted of 75 purebreds from 30 different breeds and 16 mongrels; 48 females (*Mean* age = 12.9, *SD* = 8.3; of the 48 females, 26 were neutered, for 3, there was no such information available, and the remaining 19 were not in heat at the time of the sleep recording) and 43 males (*Mean* age = 12.8, *SD* = 8.1; of the 43 males, 10 were neutered and for 1, there was no such information available).

In case of purebred dogs, actual weight was not used to index weight, as actual weight can be more of an indicator of the animals’ body condition rather than of size-driven maturational speed. Rather, we assigned to each dog the standard adult, breed-specific weight based on the Federation Cynologique Internationale/American Kennel Club database (http://www.fci.be/en/nomenclature/; https://www.akc.org/). In case of mixed breeds, we used the dog’s owner-reported 12-month-old weight (assessed when the dog reached 12 months of age). For details on sample demographics, see Supplementary Table S1.

### Ethics statement

This research was approved by the Hungarian “Animal Experiments Scientific and Ethical Committee” (PE/EA/853–2/2016) and was conducted in compliance with ARRIVE Guidelines and with Hungarian regulations on animal experimentation and Guidelines for use of animals in research, as outlined by the Association for the Study Animal Behaviour (ASAB). All owners participated voluntarily and signed an informed consent form.

### Procedure

All sleep recordings had a minimum duration of 0.5 h and a maximum duration of 3 h and were conducted after a relatively active day (e.g., mentally and physically loaded due to advanced training, excursion), during the afternoon, with a start time between 12 and 6 pm. Dogs were measured at various unfamiliar locations (i.e., camp, canine sleep laboratory, unfamiliar room at breeders’, owners’ and friends’ homes), all appropriate for basic sleep recording: constantly dark, quiet environment, mattress and reading lamp for the owner and water for the dog, available ad libitum.

Before measurements, the experimenter explained the process to the owner while the dog could explore the room (5–10 min). Prior to sleep, in case of the Juvenile sample only, owners were asked to have their dog execute a few familiar, simple tasks (e.g., lay down, give paw, sit) and praise them with treats/verbally, so as to ease any excitement the dog may have experienced in the novel environment.

After such familiarization, the owner and dog settled on the mattress and the owner gently held the dog’s head while the experimenter applied the surface electrodes. During electrode placement, dogs were rewarded using food and/or social (e.g., petting, praise) reward.

In cases where a dog did not appear calm and/or comfortable with electrode placement, the experimenter added brief breaks (~ 5 min) before continuing with electrode placement. If more than 60 min passed since arrival time and electrode placement was unsuccessful, the owner was asked to come back with the dog on another occasion. The first attempt to measure sleep EEG was unsuccessful in the case of 17 dogs, two of whom were not invited back for a second attempt due to aggressive behaviour, such as biting. Of those invited back for a second attempt (*n* = 15), the majority were measured successfully (*n* = 8). If the second attempt was also not successful for measure, the dog was excluded from the study.

After electrode placement and checking of appropriate quality of polysomnography (PSG) signals, owners were asked to mute their cell phones and, for the duration of the measurement, engage in a quiet activity such as reading, watching a movie on a laptop with earphones or sleeping. The experimenter left the room and monitored the measurement in an adjacent room. If the displacement or malfunction of an electrode occurred, the experimenter entered the sleep lab to fix it.

### EEG/PSG method

Dogs were measured between 2012 and 2020. During this period, our lab improved and updated its electrode placement and recording methods. Specifically, in our prior studies, only the frontal (Fz) channel was recorded^7,26–28^. Since then, the electrode placement has been updated and instead of one, four EEG channels were recorded^29^. Further, in the Juvenile sample, four EEG channels and an eye movement channel had been recorded (and not ECG, EMG and respiration as in case of the > 14-month-old dogs).

**Figure 1:**
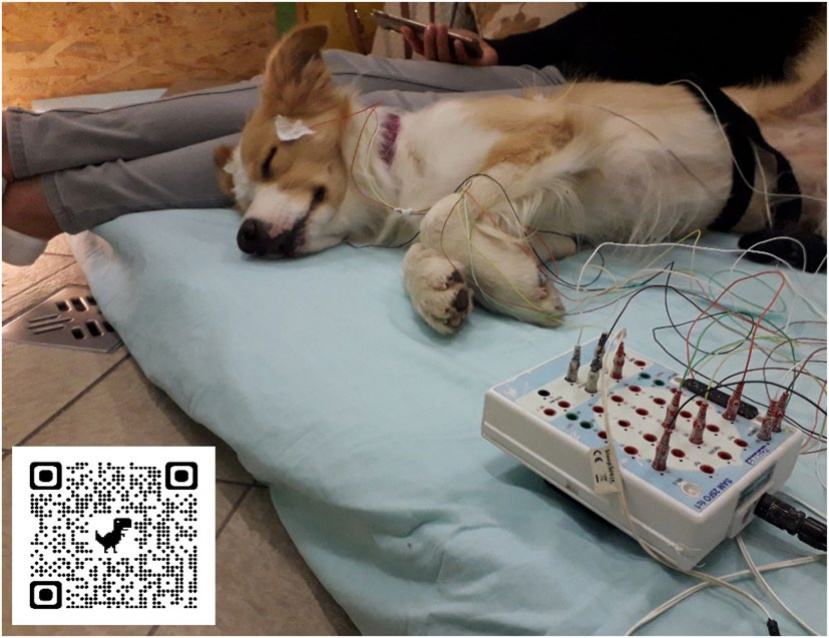
Photo of a dog with electrode placement before the sleep measurement.

The two electrodes placed on the right and left zygomatic arch next to the eyes (F8, F7) and the scalp electrodes over the anteroposterior midline of the skull (Fz, Cz) were referred to the G2, a reference electrode which was in the posterior midline of the skull (occiput; external occipital protuberance). The ground electrode (G1) was attached to the left musculus temporalis. See Fig. 1 for photo of a dog with all electrodes applied and Supplementary Fig. S1 for a schematic drawing of electrode positions on a dog’s head.

As at least the frontal electrode (Fz) was active in all dogs (i.e., in some cases of the > 14-month-old dogs), data from this electrode were used for spectral analyses. For electrode placement, Signa Spray Electrode Solution was used to separate the dog’s fur where Gold-coated Ag/AgCl electrodes were attached onto the skin using EC2 Grass Electrode Cream (Grass Technologies, USA) and impedance values were kept below 20 kΩ. As further visualisation of these electrode placement procedures, a representative video is available on our youtube channel (see QR code on Fig. 1).

Recordings were obtained with one of the following three technical arrangements.

1. In case of 47 dogs (51.6% of the total sample) the signal was collected, amplified and digitised at a sampling rate of 1000 Hz/channel, using the 40-channels NuAmps amplifier (© 2018 Compumedics Neuroscan) and DC-recording, and saved in .cnt format with the Scan 4.3 Acquire software (© 2018 Compumedics Neuroscan), converted to .edf format using MatLab EEG Toolbox.
2. In case of 37 dogs (40.7% of the total sample) the signal was collected, pre-filtered, amplified and digitised with a sampling rate of 1024 Hz/channel using a SAM 25 R style MicroMed Headbox (MicroMed Inc., Houston, TX, USA). The hardware passband was set at 0.5–256 Hz, sampling rate of 512 Hz, anti-aliasing filter with cut-off frequency at 1 kHz, and 12-bit resolution covering a voltage range of ± 2 mV as well as second-order software filters (high pass > 0.016 Hz, low pass < 70 Hz) using System Plus Evolution software (MicroMed Inc, Houston, TX, USA), which exported data in .edf format.
3. In case of 7 dogs (7.7% of the total sample) the signal was collected, pre-filtered, amplified and digitised with a sampling rate of 249 Hz/channel using a 30-channel Flat Style SLEEP La Mont Headbox with implemented second-order filters (high pass > 0.5 Hz, low pass < 70 Hz), and HBX32-SLP 32 channel pre-amplifier (La Mont Medical Inc., USA).

To correct for differences in EEG filter characteristics across recording devices, a calibration process was implemented on devices (1) and (2). Specifically, a waveform generator at the Fz electrode input of both devices was used to apply 40 and 355 μV amplitude sinusoid signals at various amplitudes (0.05 Hz, every 0.1 Hz between 0.1 and 2 Hz, every 1 Hz between 2 and 20 Hz, every 10 Hz between 10 and 100 Hz). The amplitude reduction rate for each recording system was determined by calculating the proportion of digital (measured) and analog (generated) amplitudes of sinusoid signals. Next, amplitude reduction rates were calculated for each device and EEG spectrum amplitudes were corrected by dividing such calculated values by the obtained amplitude reduction rate for the recording system. The calibration process could not be implemented on device (3), as it stopped working before the end of the project. Thus, data recorded with that device (*n* = 7 dogs) were only included in sleep macrostructure analyses, but not in EEG spectrum analyses.

### Data analysis

Both sleep macrostructure and spectral data were analysed. Sleep macrostructure variables were examined in 91 dogs (*Mean* age = 12.9, *SD* = 8.2), and spectral variables in 79 dogs (*Mean* age = 12.1, *SD* = 7.9) (this discrepancy is due to artifacts on Fz (*n* = 6) and the inability to calibrate device (3) (*n* = 7).

Sleep recordings were visually scored in accordance with standard criteria^55^, adapted for dogs^7^ and previously shown to reliably identify stages of wake, drowsiness, NREM and REM in dogs^7,56^. Data was analysed and exported using Fercio’s EEG Plus 2009–2020 software (developed by Ferenc Gombos). Sleep data consisted of 0.5–3-h-long recordings, with variability in recording length being due to differences in subject compliance. Juvenile dogs were more likely to fall asleep early (see “Results”), yet, if and when they woke up, they often became active and in such cases, recording had to be discontinued regardless of the length of elapsed time. Conversely, if and when > 14-month-old dogs woke up, they continued lying next to their owner, relaxed, and/or fell back asleep, and thus recording could be continued for the duration of 3 h. Figures S2 and Figs. S3 (see “Supplementary”) show that age was associated with time spent awake (with older dogs spending more time awake) but not with time spent asleep (minutes of drowsiness + NREM + REM sleep during the recording) (see “Results”). Given these inherent age-related differences between dogs (resulting in procedural, i.e., recording duration differences), to control for any potential biases, relative values were used in all sleep macrostructure variables (time spent in drowsiness, NREM and REM). Further, as age was associated with time spent awake, wake-related variables (e.g. sleep latency 1: elapsed minutes from the onset of recording until the first epoch scored as drowsiness; sleep latency 2: elapsed minutes from the onset of recording until the first epoch scored as NREM sleep) were excluded from analyses.

Relative power spectra were calculated only for NREM sleep, which provided most artifact-free traces and only for the Fz channel that was uniformly recorded for all dogs. Subjects with artifacts throughout the whole recording on this channel (*n* = 3 dogs) were excluded. For the remaining subjects (*n* = 57) artifact rejection of the EEG trace was carried out manually in 4 s epochs. Average power spectral values (1–30 Hz) were calculated by a mixed-radix Fast Fourier Transformation (FFT) algorithm, applied to the 50% overlapping, Hanning-tapered 4 s windows of the EEG signals of the Fz-G2 derivation. The relative power spectra were calculated as the proportion of total power (1–30 Hz), and frequency ranges of delta (1–4 Hz), theta (4–8 Hz), alpha (8–12 Hz), sigma (12–16 Hz) and beta (16–30 Hz) were used.

### Analytic plan

Data for 2–14-month-old dogs (*n* = 60) were statistically analysed and the data of older dogs (*n* = 31; 2–30 months old) were included in Figures to illustrate trends in the data across a broader age range.

Age-related differences in sleep parameters, including sleep latency, wake and sleep duration were analysed with Kendall rank correlation (because age, sleep latency and wake were non-normally distributed).

We used general additive models (GAMs)^57^ to assess possibly non-linear effects of age on sleep parameters. As an extension of the generalized linear (regression) model, in addition to linear relationships between predictors and dependent variables, non-linear terms can also be included in GAMs. As such, GAMs are more flexible than simple linear models, which makes them especially useful in analysis of developmental data^58,59^. A predefined non-linear function does not have to be specified, as the nonlinear function (i.e., smooth) is determined automatically, in any number of dimensions. The estimated degrees of freedom (edfs) provide an estimation of the complexity of the smooth, with greater edf values indicating greater complexity (e.g., edf = 1 indicates a linear association). In our model, the appropriate degree of smoothness was determined based on Restricted Maximum Likelihood (REML) to prevent overfitting. As model validation and visualization are further informative given that model values are always approximate, we also inspected (visually) our data for appropriateness of the automatically applied smooth and applied a smoothing parameter when warranted (e.g., in cases of overfitting).

GAM analyses were performed using the mgcv R package (version 3.6.3^60^) and results were plotted using the ggplot R package (version 3.6.3). GAMs were conducted to assess the effect of age, weight, and the interaction between age and weight as the independent variables (IV) (entered into GAMs as covariates) on macrostructural and spectral sleep parameters as dependent variables (DV), i.e., drowsiness, NREM and REM sleep as well as delta and theta power activity. Prior to analyses, assumptions of GAM (i.e., normality distribution of residuals) were checked. Except for NREM sleep, which was normally distributed, the DVs were positively skewed. As a result, distribution of model residuals was also non-normal. Thus, GAMs were fitted using the Gamma family (as recommended for variables with no possibility for negative values) in case of drowsiness and delta and theta power activity and Tweedie family (as recommended for variables with the possibility for zero values, but not negative values) in case of REM. Consequently, distributions of residuals were normal.

In case of covariates, collinearity and concurvity were checked. In GAMs, when there is no collinearity between covariates, an additional pitfall is if two covariates exhibit concurvity (one may be a smooth curve of another, i.e., there may be a non-linear interaction between them). Concurvity would not only increase the variance of coefficients but also enlarge their standard deviation. Age and weight showed neither collinearity (*p* = 0.93, Adj. *R*^2^ = − 0.017) nor concurvity (indicators of concurvity range from zero to one, and a value > 0.5 suggests concurvity; in our data the highest value was 0.38).

Results of statistical analyses are presented first (Juvenile sample [2–14 months]) and figures to illustrate trends in the data across a broader age range are presented second (Extended sample (2–30 months)).

## Results

In the Extended sample older age was associated with longer sleep latency (τ_b_ = 0.272, *p* < 0.001) and more wake duration (τ_b_ = 0.381, *p* < 0.001) but not with sleep duration (τ_b_ = 0.057, *p* = 0.422), (see Supplementary Figs. S2 and S3). This, combined with the resulting procedural differences (early termination of the recordings when juvenile dogs woke up and became active, see “Methods”), led us to exclude sleep latency and wake-related sleep parameters from all further analyses. Further, examples of representative hypnograms for dogs with different ages are included in our Supplementary document (Fig. S4).

### Juvenile sample (2–14 months)

#### Drowsiness, NREM and REM sleep (*n* = 60)

**Figure 2:**
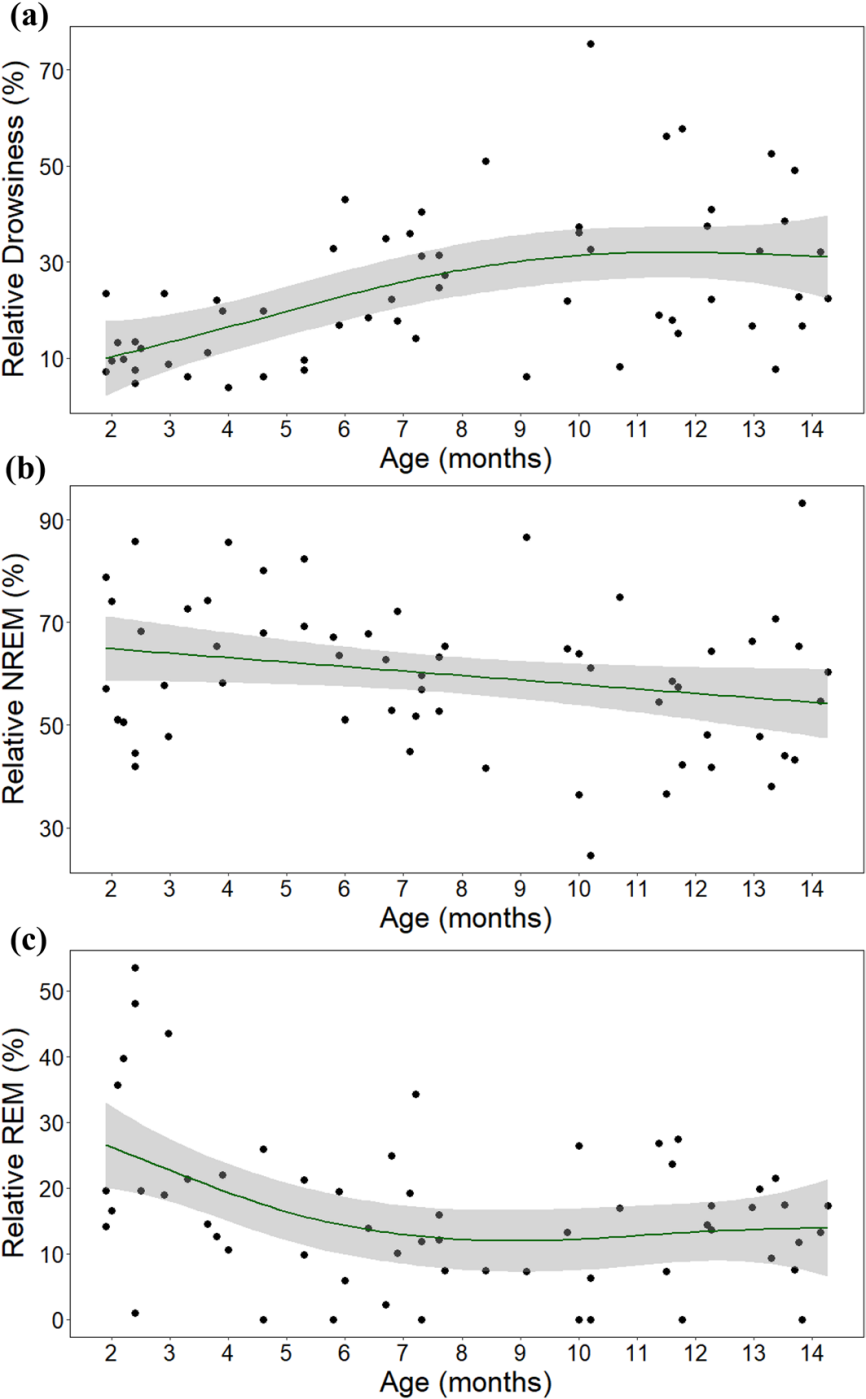
The association between age and relative duration of (**a**) drowsiness, (**b**) NREM and (**c**) REM sleep.

There was a positive association between age and relative time spent in drowsiness (*F* = 9.517, *p* < 0.001; Fig. 2a). Specifically, visual inspection indicated there was a strong positive association between these variables between the ages of 2 months to roughly 8 months and that after the age of 8 months, time spent in drowsiness sleep seemed to stabilise, though its variance increased. Weight and the interaction of age and weight had no effect (all *p*s > 0.05).

Age was not associated with relative time spent in NREM (*F* = 1.598, *p* = 0.192; Fig. 2b) and weight and the interaction between age and weight also had no effect (all *p*s > 0.05).

Age was negatively associated with relative time spent in REM (*F* = 4.363, *p* = 0.014; Fig. 2c). Visual inspection indicated that there was a negative association between these variables from the age of 2 months to roughly 6 months and that after the age of 6 months, time spent in REM seemed to stabilise. At the ages of 2–3 months, REM variance was large (Mean = 27.6%, *SD* = 16.0%). Individual differences in sleep location or weight were highly unlikely to cause this effect, as two of the five dogs that spent the most time in REM slept at the laboratory, and three of the five dogs had a standard adult weight of 35 kg (the other two had a standard adult weight of 9.5 and 23 kg) (for details see Supplementary Table S1, dogs are marked with *). Weight and the interaction of age and weight had no effect (all *p*s > 0.05). See Table 1 for a summary of macrostructural GAM results.

**Table 1:** Results of generalized additive models.

Drowsiness. Formula: drows. ~ s(age) + s(weight) + ti(age, weight)				
Parametric coefficients	Estimate	SE	*t*	*p*
Intercept	3.079	0.065	47.1	< 0.001
Smooth terms	e*df*	Ref. *df*	*F*	*p*
s (age)	2.340	2.870	9.517	< 0.001
s (weight)	1	1	0.245	0.625
ti (weight, age)	6.499	8.582	1.895	0.083
*R*^2^ (adj) = 0.461, deviance explained = 52.8%				

NREM. Formula: NREM ~ s(age, sp = 10) + s(weight) + ti(age, weight)				
Parametric coefficients	Estimate	SE	*t*	*p*
Intercept	59.679	1.751	34.09	< 0.001
Smooth terms	e*df*	Ref.*df*	*F*	*p*
s (age)	1.204	1.371	1.598	0.192
s (weight)	1	1	1.066	0.307
ti (weight, age)	5.685	7.688	1.334	0.260
*R*^2^ (adj) = 0.19, deviance explained = 29.8%				

REM. Formula: REM ~ s(age) + s(weight) + ti(age, weight)				
Parametric coefficients	Estimate	SE	*t*	*p*
Intercept	2.728	0.094	28.92	< 0.001
Smooth terms	e*df*	Ref.*df*	*F*	*p*
s (age)	2.1	2.591	4.363	0.014
s (weight)	1	1	2.543	0.117
ti (age, weight)	1.82	2.275	1.013	0.311
*R*^2^ (adj) = 0.282, deviance explained = 22.9%				

A vector of smoothing parameter (sp) was added in the GAM of NREM sleep, because in the original model the degree of smoothness was overfitted, assuming a more complex association between age and NREM due to great variance in the data.

#### Spectral analysis (in NREM sleep) (*n* = 57)

**Figure 3:**
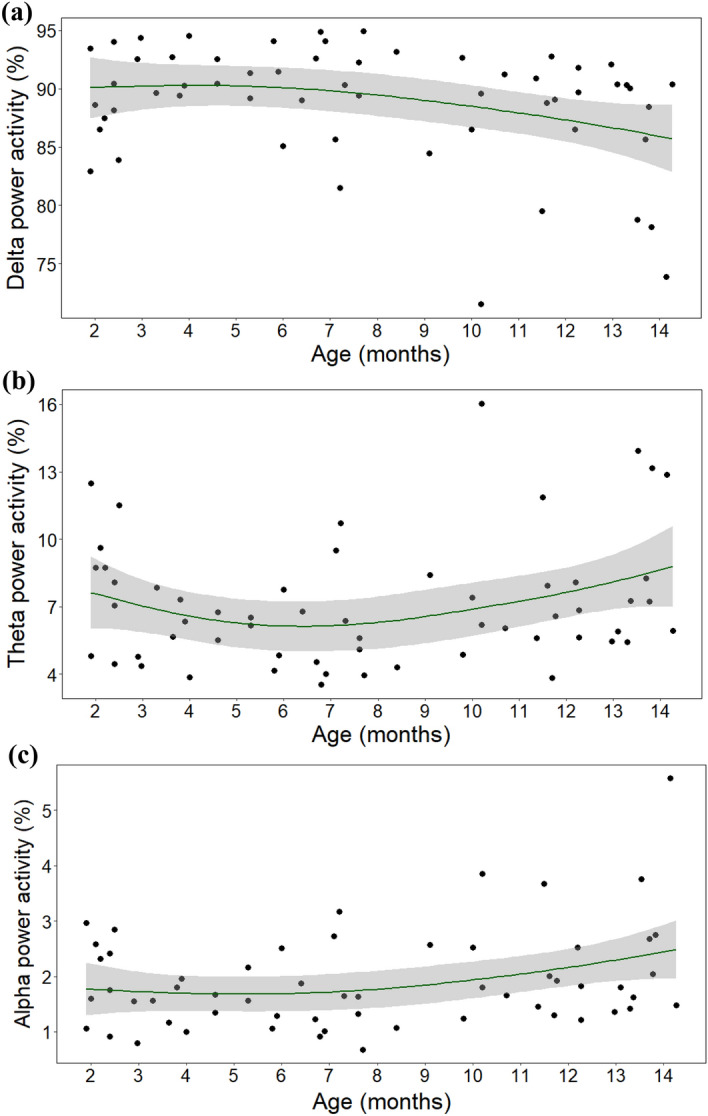
The association between age and (**a**) delta, (**b**) theta and (**c**) alpha power activity.

**Figure 4:**
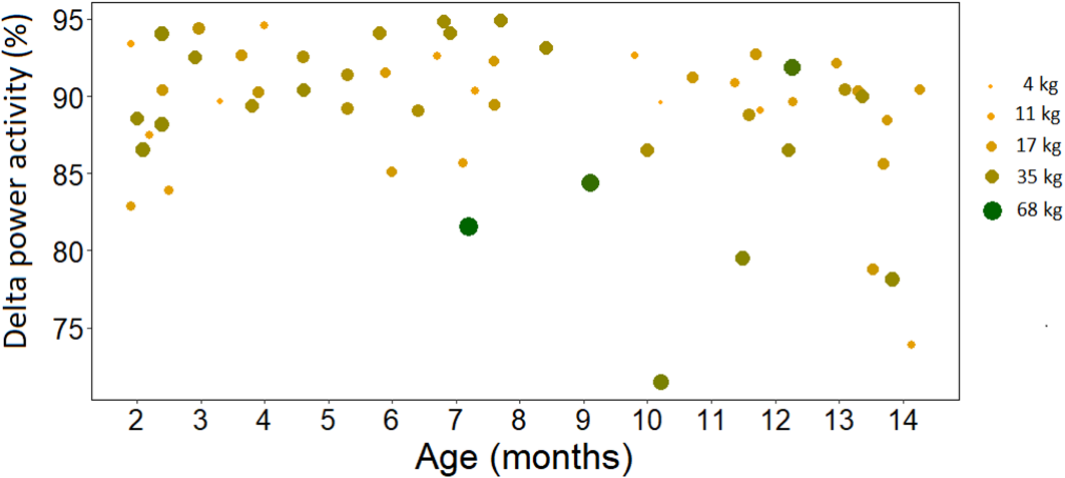
The association between age and delta power activity, given weight. Darker and larger dots indicate larger dogs.

Age was negatively associated with delta power activity (*F* = 3.337, *p* = 0.046; Fig. 3a). Specifically, visual inspection indicated that before the age of 8 months no differences in delta activity were observable but between the ages of 8 and 14 months, at greater ages, there were lower delta power values. Moreover, there was an interaction between age and weight (*F* = 2.080, *p* = 0.049, Fig. 4), with visual inspection suggesting that after the age of 8 months, larger dogs had less delta activity (weight range: 4–68 kg). Weight had no main effect (*F* = 0.103, *p* = 0.804).

Of note, in the current sample, there were *n* = 4 extra large dogs (with weights of 42.5, 55, 60.8, and 68 kg), and these were not proportionally distributed in terms of their age. To check whether the interaction effect of age and weight on delta is driven by this bias, analyses were repeated with these four dogs excluded. As previously, age was negatively associated with delta power activity (F = 7.284, *p* < 0.001), but the interaction effect between age and weight was only present at a trend level (*F* = 1.489, *p* = 0.053). Weight had no main effect (*F* = 1.489, *p* = 0.324).

Age was positively associated with theta power activity (*F* = 3.361, *p* = 0.022; Fig. 3b). Visual inspection showed that before the age of 8 months no differences in theta activity were observable but between the ages of 8 and 14 months, at greater ages, there were greater theta power values. Weight and the interaction between age and weight had no effect (all *ps* > 0.05).

Age was positively associated with alpha power activity (*F* = 3.289, *p* = 0.045; Fig. 3c). Visual inspection indicated that before the age of 8 months no differences in alpha activity were observable but between the ages of 8 and 14 months, at greater ages, there were greater alpha power values. Moreover, there was an interaction between age and weight (*F* = 2.154, *p* = 0.039), with visual inspection suggesting that after the age of 8 months, larger dogs had more alpha activity. Weight had no main effect (*F* = 1.489, *p* = 0.324). After exclusion of the four extra large dogs, analyses were repeated and the interaction effect between age and weight was no longer significant (*F* = 1.777, *p* = 0.109). As previously, age was positively associated with alpha power activity (*F* = 5.478, *p* = 0.002) and weight had no main effect (*F* = 1.418, *p* = 0.240).

**Figure 5:**
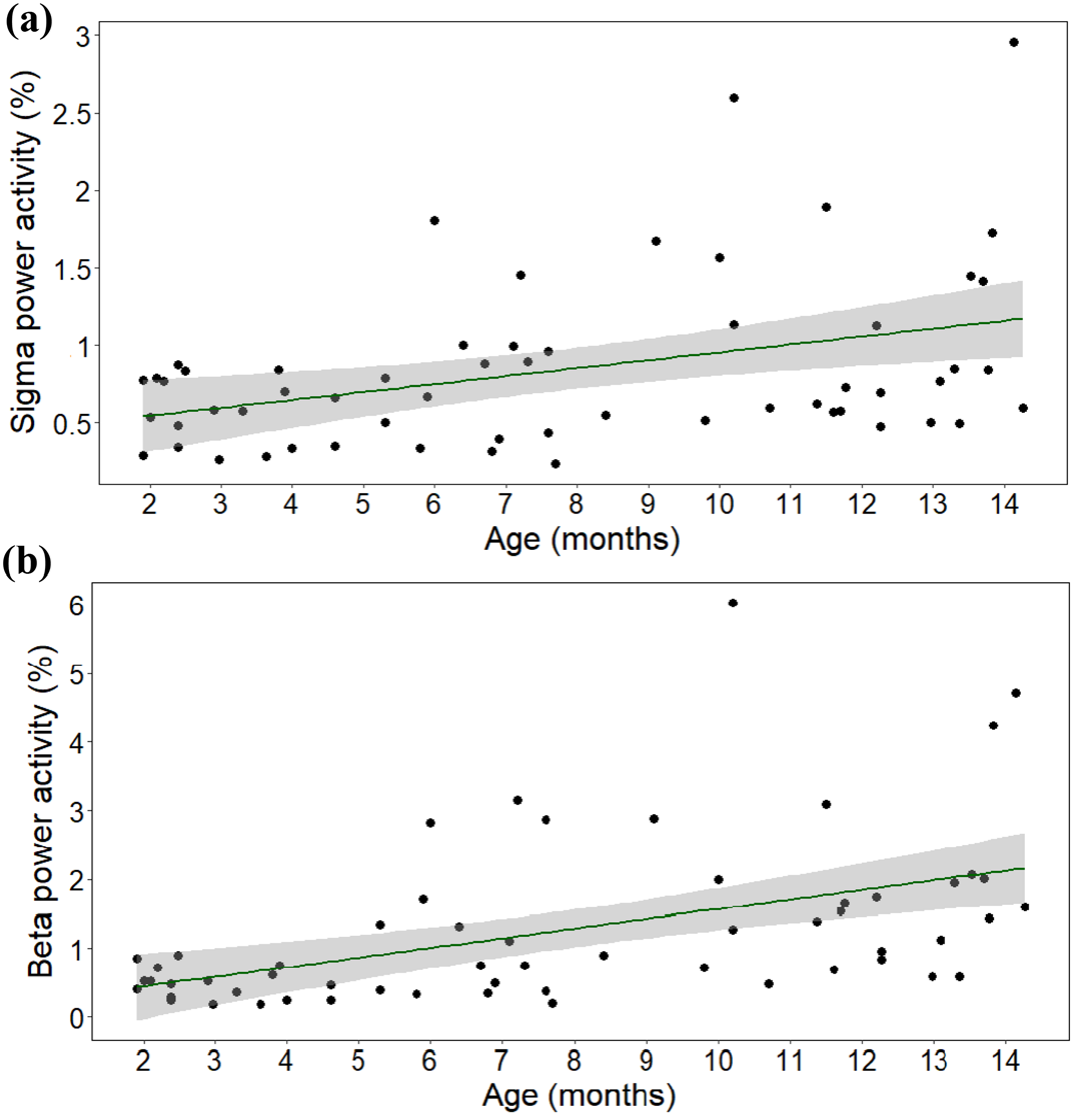
The association between age and (**a**) sigma and (**b**) beta power activity.

Age was positively associated with sigma power activity (*F* = 9.746, *p* = 0.003; Fig. 5a). Visual inspection showed a linear association, at greater ages, there were greater sigma power values. Weight and the interaction between age and weight had no effect (all *ps* > 0.05).

Age was positively associated with beta power activity (*F* = 38.333, *p* < 0.001; Fig. 5b). Visual inspection indicated that at greater ages, there were greater beta power values. Moreover, there was an interaction between age and weight (*F* = 2.495, *p* = 0.016; see Supplementary Fig. S5), with visual inspection suggesting that after the age of 8 months, larger dogs had more beta activity. Weight had no main effect (*F* = 0.123, *p* = 0.727). After exclusion of the four extra large dogs, analyses were repeated and the interaction effect between age and weight remained significant (*F* = 2.164, *p* = 0.037). As previously, age was positively associated with beta power activity (*F* = 52.186, *p* < 0.001) and weight had no main effect (*F* = 2.606, *p* = 0.114).

See Table 2 for a summary of spectral GAM results.

**Table 2:** Data of generalized additive models.

Delta. Formula: delta ~ s(age) + s(weight, sp = 10) + ti(age, weight)				
Parametric coefficients	Estimate	SE	*t*	*p*
Intercept	4.49	0.006	79.9	< 0.001
Smooth terms	e*df*	Ref. *df*	*F*	*p*
s (age)	2.142	2.625	3.337	0.045
s (weight)	1.086	1.134	0.103	0.804
ti (weight, age)	7.093	9.182	2.08	0.049
*R*^2^ (adj) = 0.36, deviance explained = 46.5%				

Theta. Formula: theta ~ s(age) + s(weight) + ti(age, weight)				
Parametric coefficients	Estimate	SE	*t*	*p*
Intercept	1.926	0.044	44.12	< 0.001
Smooth terms	e*df*	Ref. *df*	*F*	*p*
s (age)	2.672	3.275	3.361	0.022
s (weight)	1	1	0.271	0.605
ti (weight, age)	5.815	7.801	1.311	0.226
*R*^2^ (adj) = 0.26, deviance explained = 41.7%				

Alpha. Formula: alpha ~ s(age) + s(weight) + ti(age, weight)				
Parametric coefficients	Estimate	SE	*t*	*p*
Intercept	0.587	0.048	12.13	< 0.001
Smooth terms	e*df*	Ref.*df*	*F*	*p*
s (age)	2.301	2.817	3.289	0.045
s (weight)	1	1	0.250	0.619
ti (weight, age)	7.665	9.753	2.154	0.039
*R*^2^ (adj) = 0.39, deviance explained = 48.8%				

Sigma. Formula: sigma ~ s(age) + s(weight) + ti(age, weight)				
Parametric coefficients	Estimate	SE	*t*	*p*
Intercept	− 0.278	0.064	− 4.345	< 0.001
Smooth terms	e*df*	Ref. *df*	*F*	*p*
s (age)	1	1	9.746	0.003
s (weight)	1	1	0.114	0.737
ti (weight, age)	7.267	9.39	1.942	0.065
*R*^2^ (adj) = 0.39, deviance explained = 48.7%				

Beta. Formula: beta ~ s(age) + s(weight) + ti(age, weight)				
Parametric coefficients	Estimate	SE	*t*	*p*
Intercept	− 0.0379	0.079	− 0.477	0.635
Smooth terms	e*df*	Ref.*df*	*F*	*p*
s (age)	1	1	38.333	< 0.001
s (weight)	1	1	0.123	0.727
ti (weight, age)	8.114	10.27	2.495	0.016
*R*^2^ (adj) = 0.47, deviance explained = 62.4%				

To see the possible influence of body condition of mix breeds dogs on our relevant variables (delta, alpha and beta power activity), we run additional analysis with mix breed dogs excluded. For results see the “Supplementary” document.

### Extended sample (2–30 months)

**Figure 6:**
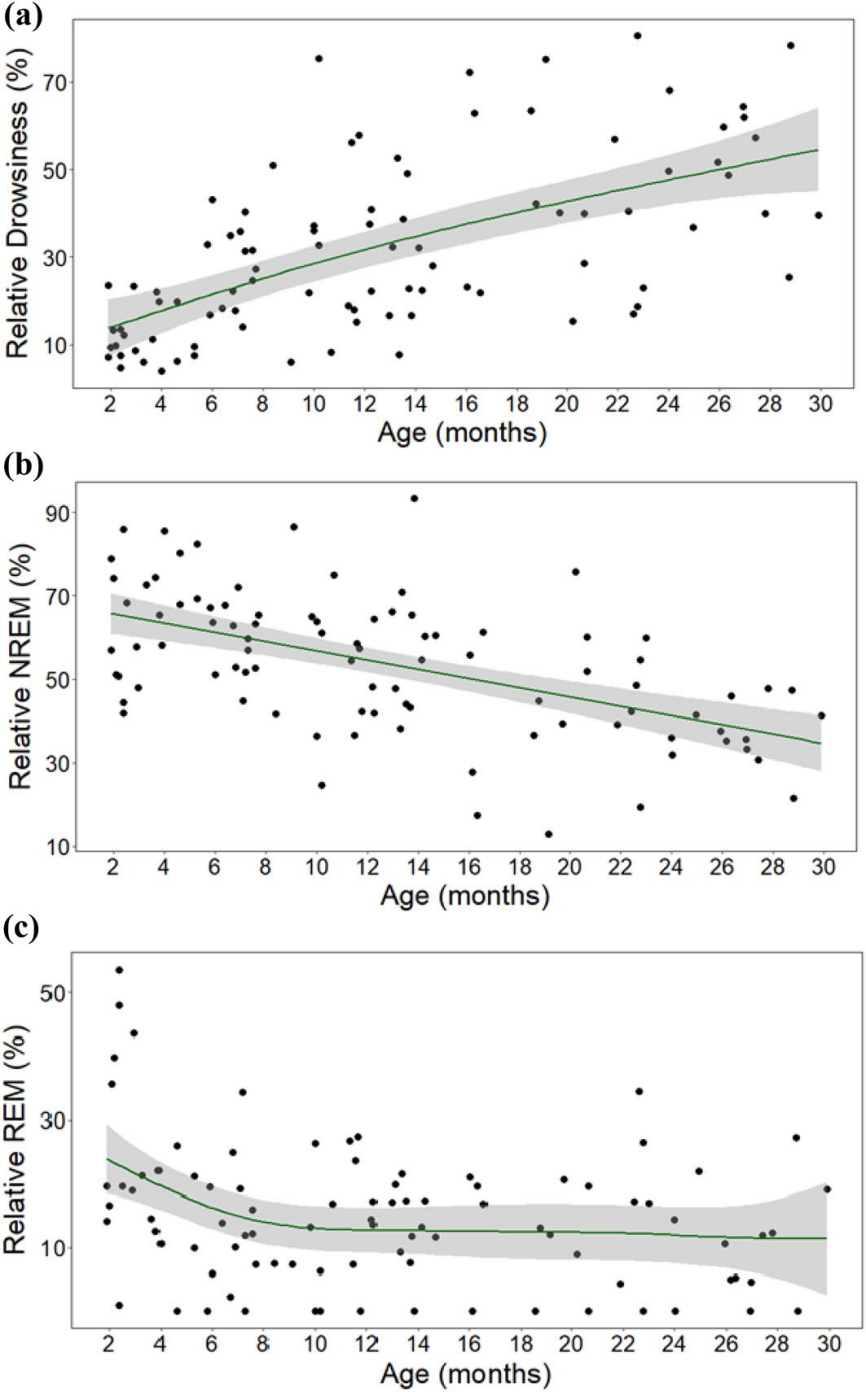
The association between age and relative duration of (**a**) drowsiness, (**b**) NREM and (**c**) REM sleep in the Extended sample.

**Figure 7:**
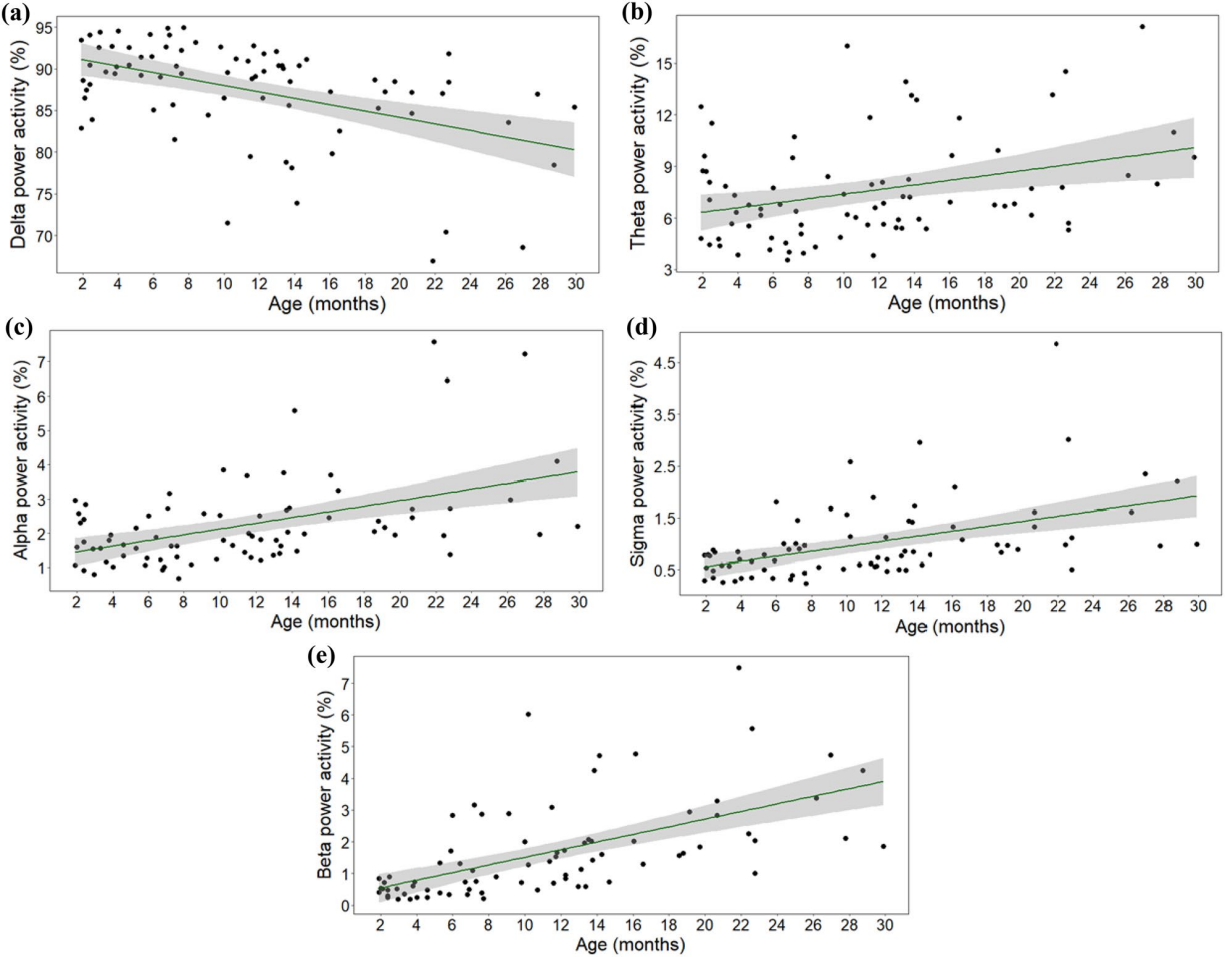
The association between age and (**a**) delta, (**b**) theta, (**c**) alpha, (**d**) sigma, (**e**) beta power activity in the Extended sample.

To illustrate trends in the data, descriptive data on sleep parameters for the Extended sample are depicted in figures (Figs. 6 and 7).

Based on visual inspection, with age, dogs seem to spend more time in drowsiness and less time in NREM, and these associations seem to be closer to linear (see Fig. 6a,b) than in the Juvenile sample. Moreover, except from more REM sleep in early life, REM appears to remain stable with age (see Fig. 6c), though it is noteworthy that 2–3-month-old dogs showed a lot of variance in this regard.

More linear associations also appear with respect to spectral values; in older dogs, there seem to be lower delta power but greater theta, alpha, sigma and beta power activity (see Fig. 7a–e).

## Discussion

This study aimed to address some gaps in knowledge via investigating family dogs’ sleep from a developmental perspective based on a large cross-sectional sample of non-invasive polysomnography data. We report associations between age and indices of sleep electrophysiology—macrostructure and power spectrum, as well as an interaction between these associations and dogs’ size (weight).

In a subset of the Juvenile sample (between the ages of 2 and 8 months) we found that older dogs spent more time in drowsiness. To our knowledge, in other non-human mammals drowsiness sleep stage was not analysed in the context of neuro-development. In human infants, NREM 1 sleep, the stage most similar to dog drowsiness^7^, is observable from the age of < 2 months^61,62^, based on the scoring suggestions of Anders et al.^63^. Relative to younger age groups (4–12 months and 13–24 months), infants between 25 and 28 months exhibit greater NREM 1 sleep^62^. However, later on in development (> 2 years, up to adolescence) age is not associated with this sleep parameter^19,64^. Taken together, these results (dog drowsiness, human NREM 1) suggest parallels between age-related differences in dog and human sleep with regard to time spent in drowsiness, in terms of older age being linked to more time in drowsiness until a certain point (human: 2 years; dog: 8 months), but there being no age-related differences in this sleep parameter thereafter (suggesting beginning of stabilisation). Earlier findings show that in humans, this stabilisation occurs during childhood, whereas the current data suggest that in dogs, it may occur at around the age of 8 months.

We did not find an association between age and the relative duration of NREM sleep. This is inconsistent with observations in the rat showing an age-related decrease in NREM beginning in adolescence^13^. Observations in humans also described a developmental decrease in slow wave sleep^19^. However, others did not find such age-related changes in NREM stage 2 nor in slow wave sleep (stage 3)^62,64^. This inconsistency may be a result of our findings with NREM including both stage 2 and slow wave sleep (stage 3), as in dogs, NREM sleep stages are not as differentiated as in humans, but, rather, constitute one stage. Of further note, there were a few dogs with very large NREM proportion values, who may have contributed to the age-NREM association being non-significant. We did not remove these animals from the analyses as there was no indication of their NREM data being inaccurate or resulting from a measurement error. As such, additional dogs with such values in a larger sample may tip the scale towards the negative age-NREM relation.

Regarding REM sleep, we observed similar age-related changes in dogs as in other species. In rats and cats^13^ as well as in humans^12,62^ there is an age-related decrease in the relative time spent in REM. In humans, this decrease stabilises around the age of 5 years^12,64^. However, others observed age-related changes in REM sleep, specifically, relative to younger and older age groups, young adults between 19 and 29 years exhibited greater REM sleep^19^. In dogs, we observed a similarly negative relation between age and REM sleep that stabilised after 6 months, which roughly corresponds to the mid-late juvenile period (i.e., between infancy and before puberty/1–12 years) in humans as indicated by research on epigenetic translation between dog and human age^65^.

In the current Juvenile sample, older dogs exhibited lower delta and higher theta, alpha, sigma and beta power activity, mostly showing the above associations evident between 8 and 14 months of age, partially in line with earlier findings. First, an age-related decrease in delta power has also been observed in invasive studies in dogs^22^ as well as in a previous non-invasive study on a sample of adult dogs (2+ years)^7^. Second, in rats and humans, slow wave activity follows an inverted U-shaped trajectory^14,20,64^. Specifically, in humans, age is positively associated with delta power during the first years of life but there is an age-related decrease in delta power from childhood to adolescence^20,64^. Higher frequencies increase in power over childhood^62,66^ and age-related differences in frequency ranges of delta, theta, alpha, sigma and beta are still present in older age groups^64^. As such, at least insofar as age-related differences in delta power go, the first 8 months in dogs may correspond to the first few years in humans. Further, brain electrophysiology in dogs between 8 and 14 months is comparable to that observed in humans in early childhood and beyond. Both sets of findings suggest human–dog age-related correspondences that are consistent with earlier estimates of such correspondence^65^.

Of further note, relative to earlier human studies with a limited number of electrodes^19,20,67^, the authors of recent studies successfully mapped changes in sleep EEG with high spatial resolution^62,64,66^. As in dog sleep EEG studies, four EEG channels are used (e.g.^29,68^), and in the current sample we analysed only the frontal EEG channel, changes in cortical topography of dog sleep could not be explored.

It has been argued that the decrease in delta power is a correlate of synaptic pruning in humans^18,21^. There is evidence of synaptic pruning in animals, such as in mice (as indexed by the number of spines within the brain and alterations in molecular signals involved in pruning^69^) and adolescent/pubertal rats (indexed by the number of the number of synaptophysin‐immunoreactive boutons^70^ and adolescent primates (e.g., *Macaca mulatta*; defined by overlapping vesicular glutamate transporter 1-positive (VGlut1+) and postsynaptic density 95-positive (PSD95+) puncta^71^), though we could not identify any studies examining whether decreased delta power is related to pruning in these species. In the current study, we have shown an age-related decrease in delta power activity in dogs and whether such decrease reflects synaptic pruning in this species is a testable hypothesis for future research.

Although there was an interaction effect between age and weight on delta, alpha and beta power activity (after the age of 8 months, larger dogs had less delta and more alpha and beta activity), this may have been driven by four large dogs that were disproportionately distributed in terms of age, as when analyses were repeated without these animals, the interaction effect became trend-level in case of delta and alpha power activity, and remained significant only in case of beta power activity. If this tendency reflects a true effect (between 8 and 14 months; weight ranged between 4 and 68 kg), it may be that at some point, these larger and thus slower-maturing animals (e.g.,^52^), begin to neurally “catch up” with their same-aged but smaller-sized counterparts, in terms of reaching or transitioning into adolescence. In addition, in the current study, we used the standard weight of pure-bred dogs, but an owner-reported weight for mongrels. It may be worth to consider the body condition of mongrels in future research, to gain a more accurate measure resembling breed standard weight.

We extended the analysis sample with data from previous dog sleep EEG studies^7,26–29^ to examine trends in when adult-like sleep parameters stabilise, if not before the age of 14 months, with the ages of the Extended sample ranging from 2 to 30 months. In the Extended sample, visual inspection showed similar associations between age and drowsiness and power spectra data as in the analysis sample, but these relations were closer to linear. In the Extended sample, visual examination indicated no association between age and REM but a negative association between age and NREM, unlike in the Juvenile sample where age was statistically associated with REM but not with NREM. This pattern across observations and results underscores the importance of careful consideration of age-related research question and sample definition; that is, the same phenomenon may manifest quite differently depending on the age-range within which it is examined, flatter or non-significant associations become steeper or significant and vice versa. In the Extended sample, visual examination further suggested that age-related differences in power spectrum do not stabilise by 14 months of age or, for that matter, even by 30 months of age. By analogy, the quantity of dog fast frontal sleep spindles also increases throughout the lifespan^35^, indicating that in dogs, certain sleep-related physiological processes mature later and their maturation continues on for longer.

## Future directions and limitations

In this study, data from single measurements were used, thus we did not examine whether a first-night effect^29^ influences the observed relations between variables. Further, short afternoon naps were measured, in locations unfamiliar to the dogs indicating our findings may not generalise to sleep at nighttime and/or at home. Determining whether the developmental patterns observed here can be replicated under different circumstances in these regards is thus a potential next step in this line of research. Nevertheless, although we had previously shown that differences in sleep parameters are associated with differences in both the timing and the location of sleep^26^, all dogs in the current study were measured in the afternoon and in unfamiliar locations, thus within-individual relations between age and sleep parameters may not be affected by these factors.

Age-related differences were measured cross-sectionally. Thus, the findings obtained in the current study are indicative of associations between age-related differences and the measured sleep parameters but not of temporal, within-animal changes over time. As such, these results indicate it is also warranted to undertake larger, longitudinal studies to assess the questions examined herein.

Regarding the interaction effect between weight and age on delta power activity, it is unclear whether the observed finding reflects a true effect or is a spurious one. The effect of weight—and of the variables weight was a proxy for—on the relation between age and delta power activity remains an area in need of further inquiry, ideally in studies that are explicitly designed to assess such effect. As the relation between heart rate and weight in dogs has been recently shown to be more complex than previously assumed^34^, the association between age, delta power activity—or sleep electrophysiology more generally—and weight may not be straightforward.

In the current research, weight was used as a proxy for differences in morphological features across dogs and differences in developmental rates between breeds. Although adult weight in dogs tends to correspond to muscle and skull thickness, there may be nuanced aspects of these characteristics that are not well approximated by weight but do impact sleep EEG. Nevertheless, the current sample was representative both with regard to head musculature and skull shape and thickness and with regard to weight, which, although prevented direct comparisons of subgroups, also excludes the possibility of any strong biases in our data that could have been due to these features.

As data trends indicated age-related differences in power spectrum may not stabilise by 14 months of age (as in humans and rats, where such differences do not stabilise by adolescence^2^—or, for that matter, not even by 30 months—there is a need to quantitatively analyse age-related differences in these variables across a broader age range. Nevertheless, based on the current findings, stabilization of neurodevelopment in the dog brain does not occur by the commonly adopted 12-month mark. This is similar to humans, where neuromaturation continues well into “young adulthood”, with gray/white matter changes continuing beyond the age of 18^72^ and white matter developing into the late 20s^73^, despite the commonly accepted 18-year cutoff of adulthood^72,73^.

A potential parallel with regard to different relations between age and indices of sleep electrophysiology depending on the specific sleep parameter examined, in humans, there is a negative association between declarative memory and REM in ~ 5-year-old children, whereas there is an increasingly stronger negative association between working memory and NREM slow bands from childhood to adolescence^74^. Establishing age-related cutoffs or transition periods with regard to the association between age, indices of sleep electrophysiology, and different cognitive domains in dogs might also be worth examining in the future.

## Conclusions

Dogs are among the most studied species, both on their own right as pet animals and as a model of human social cognition and the neural correlates and underpinnings thereof. Yet, developmental differences in dog sleep electrophysiology were previously unknown. Further, in the absence of empirical evidence, it has been (implicitly) assumed in the dog behaviour and brain function literature that dogs reach adulthood at the age of 12 months.

In this first, large-scale non-invasive study on the association between age and sleep parameters in dogs, we have shown that measured indices of sleep are a good index of neurodevelopment in dogs, with some parameters, such as drowsiness and REM sleep, stabilizing in around 6–8 months, and some, such as power spectrum, not stabilizing even by 30 months. As such, the stabilization of neurodevelopment in the dog brain is a complex matter and definitive conclusions about the age at which dogs reach adulthood cannot yet be drawn. Further, the relevant effects of differences across breed will need to be addressed. Our findings do suggest that, given robust link between developmental status of the nervous system and time spent in REM sleep—which, in dogs, stabilises around 6 months—samples including dogs younger than 6 months of age should not be conceptualised as adult samples or as homogenous with regard to maturity of the nervous system. Alternatively, if the aim is to make comparisons across age groups, those should be demarcated in light of this maturational pattern. Our results also suggest both differences and similarities in terms of age-related variation in sleep macrostructure and power spectrum across dogs and humans as well as across dogs and other model species, further underscoring the validity of the dog for modelling human sleep but also highlighting potentially important inter-species developmental differences.

## Supplementary Information

Supplementary Information.
