# Author Correction: Developmental features of sleep electrophysiology in family dogs

**DOI:** 10.1038/s41598-022-05210-1

**Published:** 2022-01-21

**Authors:** Vivien Reicher, Nóra Bunford, Anna Kis, Cecília Carreiro, Barbara Csibra, Lorraine Kratz, Márta Gácsi

**Affiliations:** 1 Department of Ethology, Institute of Biology, Eötvös Loránd University, Pázmány Péter sétány 1/C, Budapest, 1117 Hungary; 2 MTA-ELTE Comparative Ethology Research Group, Budapest, Hungary; 3 Developmental and Translational Neuroscience Research Group, Institute of Cognitive Neuroscience and Psychology, Research Centre for Natural Sciences, Budapest, Hungary; 4 Institute of Cognitive Neuroscience and Psychology, Research Centre for Natural Sciences, Budapest, Hungary

Correction to: *Scientific Reports* 10.1038/s41598-021-02117-1, published online 23 November 2021

The original version of this Article contained errors.

In Table 2, subheadings “Alpha. Formula”, “Sigma. Formula” and “Beta. Formula” were incorrectly given as “Theta. Formula”.

Incorrect:

**Table:** 

**Theta. Formula: alpha ~ s(age) + s(weight) + ti(age,weight)**				
**Parametric coefficients**	**Estimate**	**SE**	***t***	***p***
Intercept	0.587	0.048	12.13	< 0.001

**Theta. Formula: alpha ~ s(age) + s(weight) + ti(age,weight)**				
**Parametric coefficients**	**Estimate**	**SE**	***t***	***P***
Intercept	− 0.278	0.064	− 4.345	< 0.001

**Theta. Formula: beta ~ s(age) + s(weight) + ti(age,weight)**				
**Parametric coefficients**	**Estimate**	**SE**	***t***	***p***
Intercept	− 0.0379	0.079	− 0.477	0.635

Correct:

**Table:** 

**Alpha. Formula: alpha ~ s(age) + s(weight) + ti(age, weight**)				
**Parametric coefficients**	**Estimate**	**SE**	***t***	***p***
Intercept	0.587	0.048	12.13	< 0.001

**Sigma. Formula: sigma ~ s(age) + s(weight) + ti(age, weight)**				
**Parametric coefficients**	**Estimate**	**SE**	***t***	***p***
Intercept	− 0.278	0.064	− 4.345	< 0.001

**Beta. Formula: beta ~ s(age) + s(weight) + ti(age, weight)**				
**Parametric coefficients**	**Estimate**	**SE**	***t***	***p***
Intercept	− 0.0379	0.079	− 0.477	0.635

Additionally, in the section “Future directions and limitations”,

“This is similar to humans, where neuromaturation continues well into “young adulthood”, with gray/white matter changes continuing beyond the age of 18^72^ and white matter developing into the late 1920s^73^, despite the commonly accepted 18-year cutoff of adulthood^72,73^.”

now reads:

“This is similar to humans, where neuromaturation continues well into “young adulthood”, with gray/white matter changes continuing beyond the age of 18^72^ and white matter developing into the late 20s^73^, despite the commonly accepted 18-year cutoff of adulthood^72,73^.”

The original Article has been corrected.

