# Supplementary material for: Developmental features of sleep electrophysiology in family dogs
Source: Sci Rep. 2021 Nov 23;11:22760. doi: 10.1038/s41598-021-02117-1 (PMC8611005; doi:10.1038/s41598-021-02117-1)
Supplement: Supplementary file 1 — Supplementary Information. [file 41598_2021_2117_MOESM1_ESM.docx]

SUPPLEMENTARY INFORMATION to:

Developmental features of sleep electrophysiology in family dogs

Vivien Reicher^1,2*^, Nóra Bunford^1,3*^, Anna Kis^4^, Cecília Carreiro^1^, Barbara Csibra^1^, Lorraine Kratz^1^, and Márta Gácsi^1,2^

*These two authors contributed equally to this work.

^1^ Institute of Biology, Eötvös Loránd University, Department of Ethology, Budapest, Hungary

^2^ MTA-ELTE Comparative Ethology Research Group, Budapest, Hungary

^3^ Developmental and Translational Neuroscience Research Group, Institute of Cognitive Neuroscience and Psychology, Research Centre for Natural Sciences, Budapest, Hungary

^4^ Institute of Cognitive Neuroscience and Psychology, Research Centre for Natural Sciences, Budapest, Hungary

| Table 1 | | | | | | |  |
| --- | --- | --- | --- | --- | --- | --- | --- |
| *Data on individual dogs across demographic variables.* | | | | | | |  |
| Dog | Breed | Age (months) | Sex | Weight (kgs) | EEG technique | Sleep location | |
| 1 | cocker spaniel | 1.9 | male | 14.0 | 2 | breeder’s home | |
| 2 | miniature schnauzer | 1.9 | male | 7.0 | 2 | breeder’s home | |
| 3 | doberman | 2.0 | female | 33.8 | 2 | breeder’s home | |
| 4* | German shepherd | 2.1 | male | 35.0 | 2 | breeder’s home | |
| 5* | mudi | 2.2 | female | 9.5 | 2 | owner’s home (unfamiliar room) | |
| 6* | German shepherd | 2.4 | male | 35.0 | 1 | laboratory | |
| 7* | German shepherd | 2.4 | male | 35.0 | 2 | breeder’s home | |
| 8 | Nova-S. duck tolling retriever | 2.4 | female | 19.1 | 2 | laboratory | |
| 9 | cocker spaniel | 2.5 | female | 12.0 | 2 | breeder’s home | |
| 10 | labrador retriever | 2.9 | male | 32.6 | 1 | laboratory | |
| 11* | podenco Ibicenco | 3.0 | female | 23.0 | 1 | laboratory | |
| 12 | border terrier | 3.3 | female | 5.7 | 1 | laboratory | |
| 13 | border collie | 3.6 | male | 19.0 | 1 | laboratory | |
| 14 | Tervueren | 3.8 | male | 29.0 | 1 | laboratory | |
| 15 | rough collie | 3.9 | female | 22.5 | 1 | laboratory | |
| 16 | dachshund | 4.0 | male | 9.0 | 1 | laboratory | |
| 17 | boxer | 4.6 | male | 32.6 | 1 | laboratory | |
| 18 | golden retriever | 4.6 | female | 27.0 | 1 | laboratory | |
| 19 | Australian shepherd | 5.3 | male | 25.9 | 1 | laboratory | |
| 20 | golden retriever | 5.3 | female | 27.0 | 1 | laboratory | |
| 21 | labrador retriever | 5.8 | female | 28.1 | 1 | laboratory | |
| 22 | mongrel | 5.9 | female n. | 15.0 | 1 | laboratory | |
| 23 | border collie | 6.0 | female | 17.0 | 1 | laboratory | |
| 24 | German shepherd | 6.4 | female | 27.0 | 1 | laboratory | |
| 25 | Boston terrier | 6.7 | male | 9.2 | 1 | laboratory | |
| 26 | golden retriever | 6.8 | male n. | 31.5 | 1 | laboratory | |
| 27 | golden retriever | 6.9 | male | 31.5 | 1 | laboratory | |
| 28 | cocker spaniel | 7.1 | female | 12.0 | 1 | laboratory | |
| 29 | tosa | 7.2 | female n. | 68.0 | 2 | laboratory | |
| 30 | mongrel | 7.3 | female n. | 9.0 | 1 | laboratory | |
| 31 | small Munsterlander | 7.3 | female | 24.0 | 1 | laboratory | |
| 32 | American staffordshire terrier | 7.6 | female | 22.1 | 1 | laboratory | |
| 33 | border collie | 7.6 | female | 17.0 | 1 | laboratory | |
| 34 | golden retriever | 7.7 | male | 31.5 | 1 | laboratory | |
| 35 | golden retriever | 8.4 | male | 31.5 | 1 | laboratory | |
| 36 | Caucasian shepherd | 9.1 | male | 60.8 | 1 | laboratory | |
| 37 | dachshund | 9.8 | female n. | 8.0 | 1 | laboratory | |
| 38 | mongrel | 10.0 | female n. | 12.5 | 1 | laboratory | |
| 39 | mongrel | 10.0 | female n. | 28.0 | 2 | laboratory | |
| 40 | miniature German pinscher | 10.2 | male n. | 4.0 | 1 | laboratory | |
| 41 | cane corso | 10.2 | female N/A | 42.5 | 1 | laboratory | |
| 42 | border collie | 10.7 | male | 19.0 | 1 | laboratory | |
| 43 | mongrel | 11.4 | male n. | 11.4 | 2 | laboratory | |
| 44 | mongrel | 11.5 | male | 38.3 | 2 | laboratory | |
| 45 | Tervueren | 11.6 | female n. | 25.0 | 1 | laboratory | |
| 46 | mongrel | 11.7 | male | 19.0 | 1 | laboratory | |
| 47 | mongrel | 11.8 | female n. | 8.2 | 2 | laboratory | |
| 48 | mongrel | 12.2 | male n. | 32.0 | 1 | laboratory | |
| 49 | cocker spaniel | 12.3 | male n. | 14.0 | 2 | laboratory | |
| 50 | Moscow watchdog | 12.3 | female | 55.0 | 2 | laboratory | |
| 51 | border collie | 13.0 | female n. | 17.0 | 2 | laboratory | |
| 52 | golden retriever | 13.1 | female n. | 27.0 | 2 | laboratory | |
| 53 | border collie | 13.3 | male | 19.0 | 1 | laboratory | |
| 54 | golden retriever | 13.4 | male | 31.5 | 2 | laboratory | |
| 55 | border collie | 13.5 | male | 19.0 | 2 | laboratory | |
| 56 | mongrel | 13.7 | female n. | 18.5 | 2 | laboratory | |
| 57 | border collie | 13.8 | female | 17.0 | 2 | laboratory | |
| 58 | white Swiss shepherd | 13.8 | male | 35.0 | 2 | laboratory | |
| 59 | dachshund | 14.1 | female n. | 11.0 | 2 | laboratory | |
| 60 | border collie | 14.3 | female | 17.0 | 2 | laboratory | |
| 61 | border collie | 14.7 | female n. | 17.0 | 2 | laboratory | |
| 62 | Hungarian vizsla | 16.0 | female n. | 23.0 | 2 | laboratory | |
| 63 | mudi | 16.1 | male n. | 12.0 | 2 | friend’s home (unfamiliar room) | |
| 64 | border collie | 16.3 | male | 19.0 | 3 | laboratory | |
| 65 | springer spaniel | 16.6 | female | 19.1 | 2 | laboratory | |
| 66 | labrador retriever | 18.6 | female n. | 28.1 | 2 | laboratory | |
| 67 | bichon Havanese | 18.8 | female n. | 6.0 | 2 | laboratory | |
| 68 | mongrel | 19.1 | male n. | 13.0 | 2 | laboratory | |
| 69 | German shepherd | 19.7 | female n. | 27.0 | 2 | laboratory | |
| 70 | mongrel | 20.2 | male | 31.5 | 3 | laboratory | |
| 71 | border collie | 20.7 | male | 19.0 | 2 | laboratory | |
| 72 | Bordeaux dog | 20.7 | male | 58.0 | 2 | laboratory | |
| 73 | mongrel | 21.9 | male n. | 32.0 | 2 | laboratory | |
| 74 | Australian shepherd | 22.4 | female n. | 21.2 | 2 | laboratory | |
| 75 | Hungarian vizsla | 22.6 | male | 25.9 | 2 | laboratory | |
| 76 | Chinese crested | 22.8 | male n. | 6.0 | 2 | hotel room | |
| 77 | golden retriever | 22.8 | female n. | 27.0 | 2 | laboratory | |
| 78 | Shetland sheepdog | 23.0 | male | 9.0 | 2 | laboratory | |
| 79 | golden retriever | 24.0 | male N/A | 31.5 | 3 | laboratory | |
| 80 | border terrier | 24.0 | female | 5.7 | 3 | laboratory | |
| 81 | Moscow watchdog | 25.0 | female N/A | 55.0 | 2 | laboratory | |
| 82 | mongrel | 25.9 | female N/A | 15.0 | 3 | laboratory | |
| 83 | Spanish water dog | 26.2 | male n. | 20.0 | 2 | laboratory | |
| 84 | mongrel | 26.4 | male n. | 30.0 | 3 | laboratory | |
| 85 | puli | 26.9 | female n. | 11.5 | 3 | laboratory | |
| 86 | Australian shepherd | 27.0 | male n. | 25.9 | 2 | laboratory | |
| 87 | Hungarian vizsla | 27.4 | male | 25.9 | 2 | laboratory | |
| 88 | mongrel | 27.8 | female n. | 28.0 | 1 | laboratory | |
| 89 | border collie | 28.7 | female n. | 17.0 | 2 | laboratory | |
| 90 | American staffordshire terrier | 28.8 | female n. | 22.1 | 2 | laboratory | |
| 91 | mongrel | 29.9 | female n. | 14.0 | 2 | laboratory | |
| *Note.* The ordering of subjects is according to ascending age. Dogs 1-60 are included in the Young sample and all dogs (1-91) are included in the Extended sample. Age was calculated as (birth date - recording date)/30. Weight was calculated based on the Federation Cynologique Internationale/ American Kennel Club database for purebred dogs and was based on owner-report for mixed breed dogs. The numbers in case of EEG techniques indicate the three technical arrangements with which recordings were obtained: (1) NuAmps amplifier, (2) SAM 25 R MicroMed Headbox, (3) Flat Style SLEEP La Mont Headbox.  *=young dogs with great variability regarding time spent in REM sleep (see Results). n.=neutered, N/A=no information on neutered/spayed status. | | | | | | |  |

**
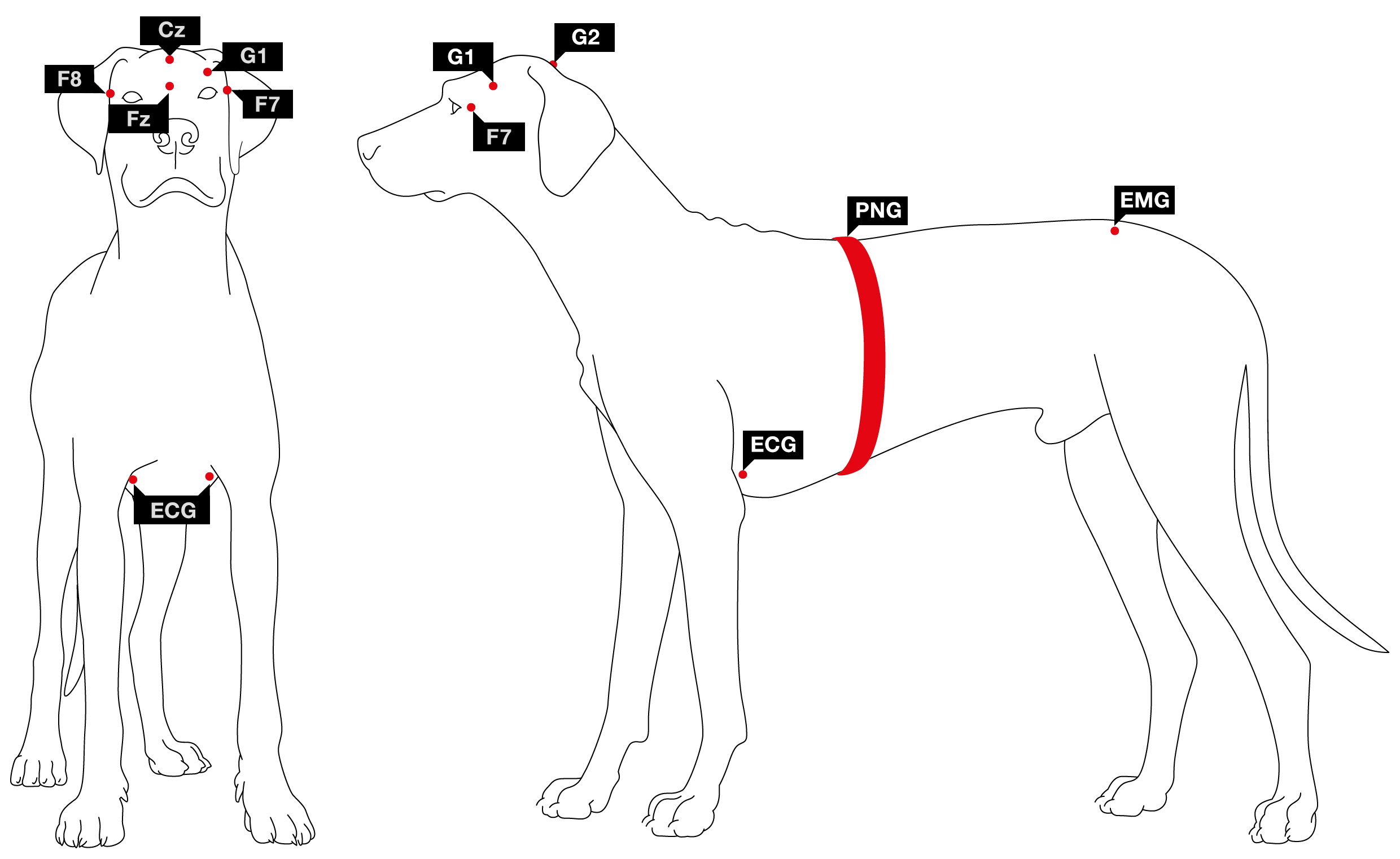
**

**Figure S1**. Electrode placement (Fz-Cz: frontal and central midline; F8 and F7: right and left electrodes placed on the zygomatic arch; G2: reference electrode; G1: ground electrode).


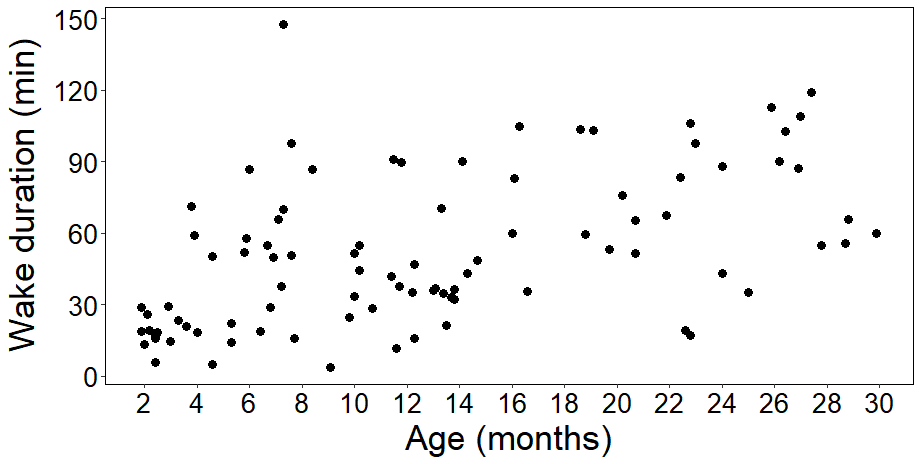


**Figure S2**: Time spent awake during the entire sleep recording across ages (Extended sample).


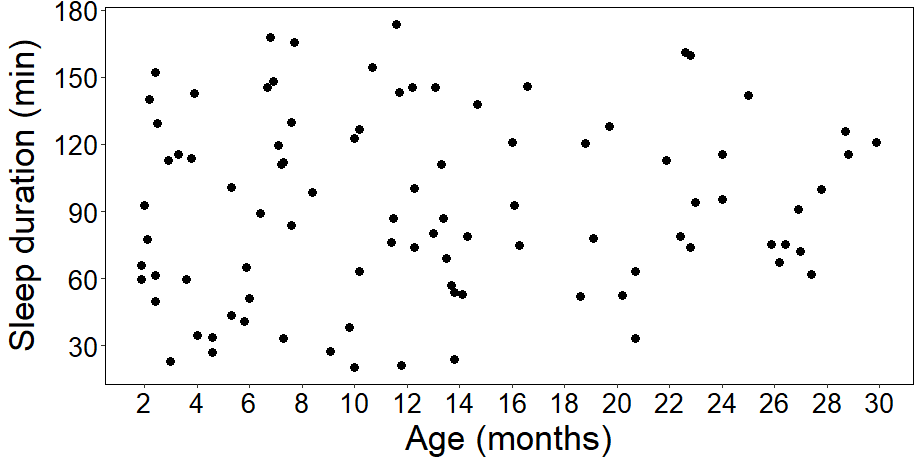


**Figure S3**: Time spent asleep during the entire sleep recording across ages (Extended sample).


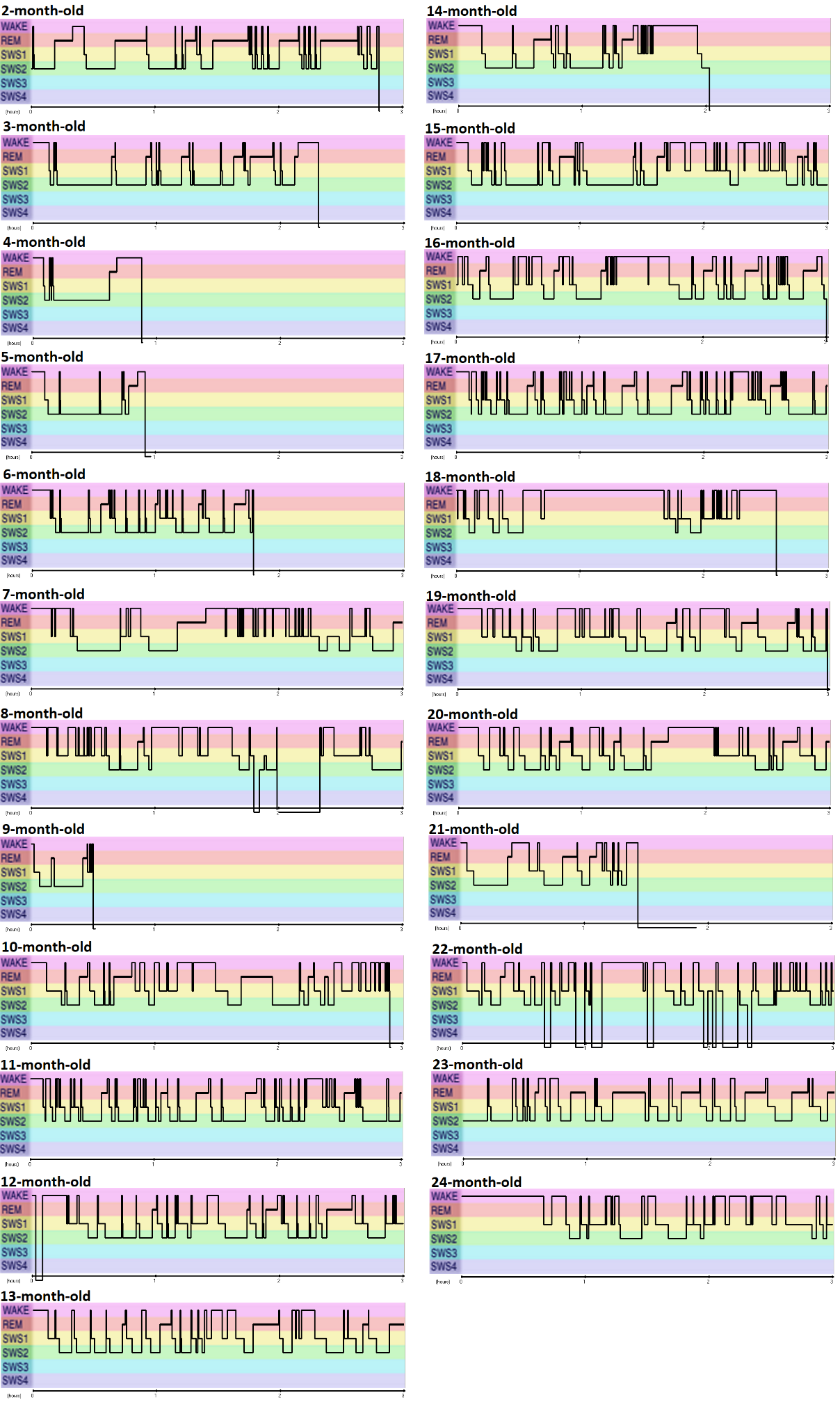


**Figure S4**: Example hypnograms of dogs from different ages. SWS1 indicates drowsiness, SWS2 indicates NREM sleep. SWS3 and 4 are not present in dogs.


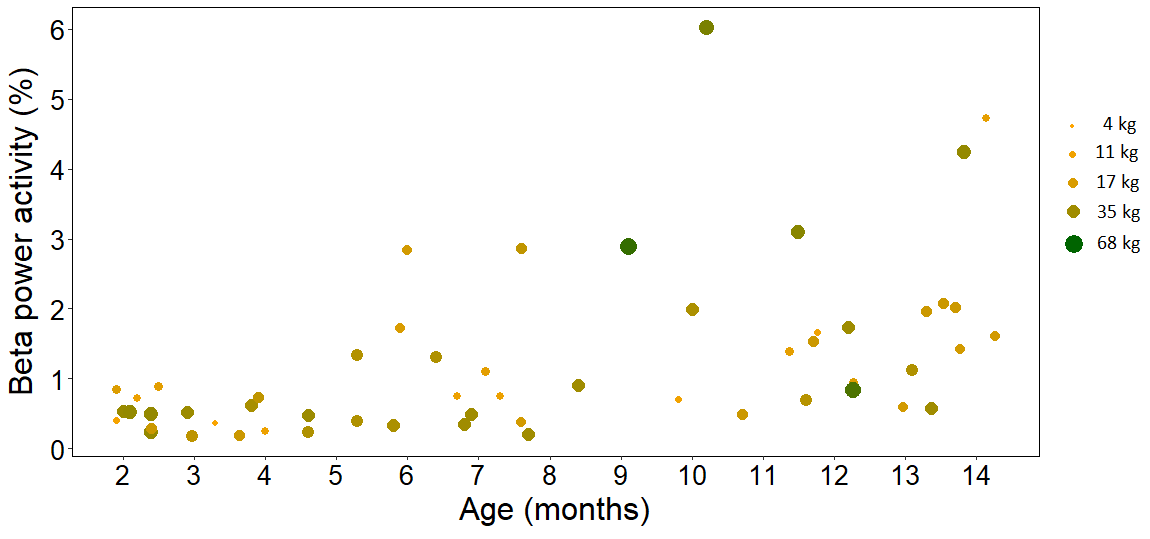


**Figure S5**: The association between age and beta power activity given weight. Darker and larger dots indicate larger dogs.

Spectral analysis excluding mix breed dogs (*n*=48):

Further, we examined the interaction effect of age and weight on delta, alpha and beta power activity without mix breed dogs (*n*=9) to control for possible effects of their body condition. With this subsample, age, weight and the interaction of age and weight had no effect on delta and alpha power activity (all *p*s>0.05). Regarding beta power activity, age was positively associated with beta power activity (*F*=29.725, *p*<.001) and weight and no effect (*F*=0.206, *p*=.653).
